# Non-selective regulation of peroxide and superoxide resistance genes by PerR in *Campylobacter jejuni*

**DOI:** 10.3389/fmicb.2015.00126

**Published:** 2015-02-17

**Authors:** Jong-Chul Kim, Euna Oh, Sunyoung Hwang, Sangryeol Ryu, Byeonghwa Jeon

**Affiliations:** ^1^School of Public Health, University of AlbertaEdmonton, AB, Canada; ^2^Department of Food and Animal Biotechnology, Department of Agricultural Biotechnology, Center for Food and Bioconvergence, Seoul National UniversitySeoul, South Korea

**Keywords:** *Campylobacter*, oxidative stress, PerR

## Abstract

*Campylobacter jejuni* is an important foodborne pathogen. The molecular mechanisms for the regulation of oxidative stress resistance have not yet been understood fully in this bacterium. In this study, we investigated how PerR (peroxide stress regulator) modulates the transcriptional regulation of both peroxide and superoxide resistance genes in *C. jejuni*, particularly under oxidative stress conditions. The transcriptional levels of *ahpC*, *katA*, and *sodB* were substantially increased by aeration and oxidant exposure. Interestingly, a *perR* mutation completely abrogated the transcriptional response of *ahpC*, *katA* and *sodB* to oxidants. Furthermore, we demonstrated that *perR* transcription was reduced by aeration and oxidant exposure. In contrast to the unique role of PerR homologs in peroxide stress regulation in other bacteria, *C. jejuni* PerR directly regulates the transcription of *sodB*, the most important gene in superoxide defense, as evidenced by the alteration of *sodB* transcription by the *perR* mutation and direct binding of rPerR to the *sodB* promoter. In addition, we also observed notable morphological changes in *C. jejuni* from spiral rods to cocoid morphology under aerobic conditions. Based on the intracellular ATP levels, *C. jejuni* entered a viable-but-non-culturable (VBNC) state under aerobic conditions. These findings clearly demonstrate that *C. jejuni* possesses a unique regulatory mechanism of oxidative stress defense that does not specifically distinguish between peroxide and superoxide defense, and PerR plays a pivotal role in this non-selective regulation of oxidative stress resistance in *C. jejuni*.

## Introduction

*Campylobacter jejuni* is one of the leading bacterial causes of gastroenteritis worldwide (Young et al., 2007). *C. jejuni* infection also accounts for the majority (approximately one quarter) of disease cases of Guillain-Barré syndrome, an acute peripheral neuropathy, as a post-infection complication (Hughes and Cornblath, 2005). *C. jejuni* is a commensal bacterium in the intestines of poultry that do not develop any clinical symptoms (Moore et al., 2005); thus, human infections with *C. jejuni* are frequently caused by the consumption of contaminated poultry (Allos, 2001). *C. jejuni* requires low oxygen concentrations (3 ~ 15%) for growth and is sensitive to high oxygen tension under normal atmospheric conditions. *C. jejuni* favorably inhabits the gastrointestinal tracts in poultry that provide optimal growth temperatures (about 42°C), nutrients, and low oxygen levels. Once excreted from animals, however, *C. jejuni* encounters various harsh environmental stress, such as high oxygen tension and nutrient starvation. Particularly, increased oxidative stress in the atmosphere is a critical barrier that *C. jejuni* should overcome during its zoonotic transmission from animals (i.e., poultry) to humans via food. Therefore, a better understanding of the molecular mechanisms of oxidative stress is critical for elucidating the infection process of this microaerophilic foodborne pathogen.

*C. jejuni* has a unique oxidative stress defense system. For the detoxification of reactive oxygen species (ROS), for example, *C. jejuni* possesses only single copies of alkyl hydroperoxide reductase (*ahpC*), catalase (*katA*), and superoxide dismutase (*sodB*) (Atack and Kelly, 2009), whereas other bacteria often harbor redundant types of these antioxidant genes. *Escherichia coli* encodes three *sod* genes (*sodA*, *sodB*, and *sodC*) and two catalase genes (*katE* and *katG*) (Imlay, 2008). Alkyl hydroperoxide reductase in *E. coli* consists of two subunits, AhpC and AhpF (Poole et al., 2000), whereas *ahpF* homologs are absent from the *C. jejuni* genome (Baillon et al., 1999). *C. jejuni* also has unique regulatory mechanisms for oxidative stress resistance. In *E.coli* and *Salmonella*, OxyR and SoxRS regulate expression of peroxide and superoxide defense regulons, respectively (Imlay, 2008; Chiang and Schellhorn, 2012). However, *C. jejuni* lacks homologs of SoxRS and OxyR (Atack and Kelly, 2009). Instead, *C. jejuni* posseses PerR, a substitute for OxyR found in many Gram-positive bacteria and some Gram-negative bacteria; in fact, *C. jejuni* is the first Gram-negative bacterium that is known to harbor PerR (Van Vliet et al., 1999). In *C. jejuni*, PerR inactivation significantly increases the expression of peroxide resistance enzymes, such as AhpC and KatA, and renders *C. jejuni* hyper-resistant to H_2_O_2_ (Van Vliet et al., 1999).

PerR belongs to the ferric uptake regulator class of metal-responsive repressor proteins (Van Vliet et al., 1999; Lee and Helmann, 2007). PerR uses either manganese or iron as a regulatory metal-cofactor to detect oxidative stress in *Bacillus subtilis* (Lee and Helmann, 2006; Jacquamet et al., 2009), whereas iron, but not manganese, affects *perR* transcription by PerR autoregulation in *C. jejuni* (Kim et al., 2011). Although PerR is the key regulator of oxidative stress defense in *C. jejuni*, the regulatory mechanism of PerR has not been fully defined in this microaerophilic foodborne pathogen, particularly under oxidative stress (e.g., aerobic conditions). In this study, we demonstrated that PerR controls the transcription of *sodB*, the most important gene in superoxide resistance, and plays a critical role in non-selective regulation of peroxide and superoxide resistance genes in *C. jejuni*.

## Materials and methods

### Bacterial strains and growth conditions

*C. jejuni* NCTC 11168 and its derivatives were grown at 42°C on Mueller-Hinton media (Difco) under microaerobic conditions (5% O_2_, 10% CO_2_, 85% N_2_). Kanamycin (50 μg/ml) was occasionally added to the culture media where required. Broth cultures were grown by shaking at 200 rpm aerobically or microaerobically.

### Quantitation of intracellular ATP levels

The intracellular ATP levels were measured by using the ATP Bioluminescent Assay Kit (Sigma) according to the manufacturer's instructions. Briefly, after washing with PBS (pH 7.4) twice, the *C. jejuni* suspension was mixed with the same volume of the ATP assay solution in a 96-well plate. After incubation at room temperature for 3 min, the luminescence was measured with FLUOstar Omega (BMG Labtech). The total protein concentrations of the sample were measured by using the Bradford assay (Bradford, 1976) to normalize the ATP levels to the protein amounts. The experiments were repeated three times, and each set was run in triplicate.

### Fluorescence microscopic analysis

*C. jejuni* was harvested after 4 h, 8 h, and 24 h culture under aerobic or microaerobic conditions and was stained with the LIVE/DEAD *Bac*Light™ bacteria viability kit (Life Technologies). Images were visualized with a fluorescence microscope (Carl Zeiss M1) and were analyzed with Axio Vision LE (Zeiss).

### Construction of *lacZ* transcriptional fusion and β-galactosidase assay

The promoters and partial coding regions of *ahpC*, *katA*, and *sodB* were PCR-amplified with the primer pairs of ahpC_PF_F(*Xba*I) and ahpC_PF_R(*Xba*I), katA_PF_F(*Xba*I) and katA_PF_R(*Xba*I), and sodB_PF_F(*Xba*I) and sodB_PF_R(*Xba*I), respectively (Table 1). The PCR products were cloned into an *Xba*I site of pMW10 (Wösten et al., 1998) that contains a promoterless *lacZ* gene. Each fusion plasmid was mobilized to *C. jejuni* NCTC 11168 by conjugation. *C. jejuni* strains harboring a *P_perR_*-*lacZ* plasmid and a *perR* complementation strain were described in our previous study (Kim et al., 2011). Occasionally, the assays were performed with Minimum Essential Medium alpha (MEMα) (Life Technologies), which does not contain iron, in the presence or absence of 40 μM FeSO_4_ (Sigma). To assess the effect of oxidative stress on the promoter activities of *ahpC*, *katA*, and *sodB*, β-galactosidase assays were carried out with oxidants, such as H_2_O_2_, cumene hydroperoxide (CHP, an organic peroxide), and menadione (MND, a superoxide generator). Briefly, *C. jejuni* in the exponential phase was harvested and resuspended in fresh MH broth to a cell concentration of 10^9^ CFU/ml, and was exposed to oxidants for 2 h prior to the assay.

**Table 1:** **Primers used in this study**.

**Primer**	**DNA sequence from 5′ to 3′**
ahpC_PF_F	TCTTCACCTTCTAGATTGTTAGTATCATC
ahpC_PF_R	CGCTGGAGCAGTAAAATCTAGAGC
sodB_PF_F	AGTAATGCTGAGTCTAGAACAACTTTTTTC
sodB_PF_R	TCCATGATGATATCTAGAAGTTTCAGC
katA_PF_F	TAAAACAGCTCTAGAAGGAGTGATTTC
katA_PF_R	TGAATTTTGGTTATCATCTAGAATGTTTCC
SB_P	GCGAAGGATCCTAGTAATGCTGAGATTAGTA
SB_R	AGAATACGAATAGCTTTTTGATAT

### Purification of recombinant PerR protein (rPerR)

Purification of rPerR was performed as described in our pervious study (Kim et al., 2011). Briefly, an *E. coli* strain carrying pET15b::*perR* was grown in Luria-Bertani (LB) broth to an optical density at 600 nm of 1.0 at 37°C, and 1 mM IPTG (isopropyl-β-D-thiogalactopyranoside) was then added. After 5 h induction at 37°C, cells were harvested and resuspended in lysis buffer (20 mM Tris-HCl [pH 8.0], 300 mM NaCl). Bacterial cells were lysed by sonication and purified under native conditions using Ni^2+^ affinity chromatography (Qiagen). The His-tag was removed from rPerR with the Thrombin Cleavage Capture Kit (Novagen) according to the manufacturer's instructions.

### Electrophoretic mobility shift assay (EMSA)

EMSA was performed as described previously with some modifications (Jeon and Zhang, 2007). The Cy3-labeled DNA probes were PCR-generated with the primers of SB-P and SB-R (Table 1). The DNA probe was incubated with rPerR at different concentrations at 37°C for 15 min in 10 μl of gel-shift assay buffer (20 mM HEPES [pH 7.6], 1 mM EDTA, 10 mM (NH_4_)_2_SO_4_, 5 mM DTT, 0.2% Tween 20, 30 mM KCl, 0.1 μg poly dI-dC). The reaction mixtures were resolved in a 6% polyacrylamide gel, and the Cy3-labeled DNA fragments were visualized with FluorChem™-R (ProteinSimple, USA).

## Results

### Differential induction of *ahpC*, *katA*, and *sodB* transcription under aerobic conditions

**Figure 1:**
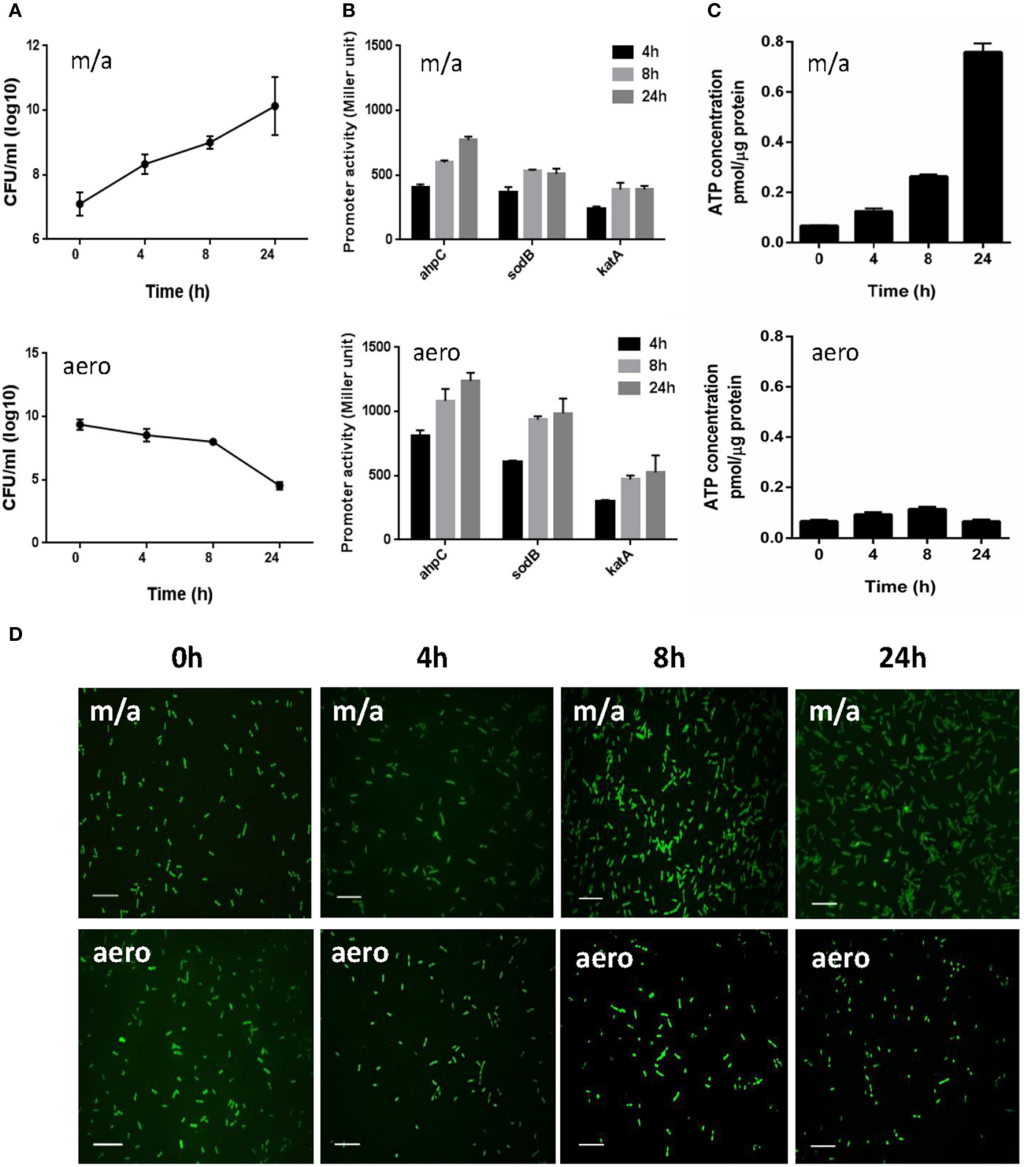
**Bacterial growth, transcriptional levels of ROS-detoxification genes, intracellular ATP levels, and morphological changes in *C. jejuni* under microaerobic and aerobic conditions**. **(A)** The growth kinetics of *C. jejuni* under microaerobic and aerobic culture conditions. The means and standard deviations of triplicate samples in a single experiment are indicated. The experiments were repeated three times. **(B)** Transcriptional levels of *ahpC*, *katA*, and *sodB* genes under microaerobic and aerobic culture conditions. *C. jejuni* strains harboring *P_ahpC_-lacZ*, *P_katA_-lacZ*, and *P_sodB_-lacZ* were grown in MH medium microaerobically or aerobically. The results are representative of at least three independent experiments. Each experiment was performed with triplicate samples and all the repeated experiments exhibited similar results. **(C)** Intracellular ATP levels in *C. jejuni* under microaerobic and aerobic conditions. The ATP concentrations were normalized to the total protein concentrations. The experiment was performed in triplicate and was repeated three times. Error bars indicate the standard deviations. **(D)** Fluorescent microscopic images of *C. jejuni* with LIVE/DEAD *Bac*Light™ staining under microaerobic and aerobic conditions. The scale bar shows 10 μm. “m/a” and “aero” indicate microaerobic and aerobic conditions, respectively.

AhpC, SodB, and KatA are the sole alkyl hydroperoxide reductase, superoxide dismutase (SOD), and catalase in *C. jejuni*, respectively, significantly contributing to oxidative stress defense in *C. jejuni* (Atack and Kelly, 2009). Although *C. jejuni* is a microaerophilic bacterium, it harbors a certain level of aerotolerance (Gundogdu et al., 2011). Due to the importance of *ahpC*, *katA*, and *sodB* in ROS detoxification, we hypothesized that *C. jejuni* would differentially regulate *ahpC*, *katA*, and *sodB* expression to scavenge toxic ROS under aerobic conditions. To measure the transcriptional levels of *ahpC*, *katA*, and *sodB*, we constructed transcriptional fusions of the *ahpC*, *katA*, and *sodB* promoters to a promoterless *lacZ* in pMW10 (Wösten et al., 1998). Although *C. jejuni* growth was significantly decreased under aerobic conditions (Figure 1A), interestingly, the transcriptional levels of *ahpC* and *sodB* were increased under aerobic conditions (Figure 1B). After 24 h culture under aerobic conditions, the promoter activities of *ahpC* and *sodB* were increased by approximately 62% and 89%, respectively, compared to microaerobic conditions (Figure 1B).

Despite a significant growth defect under aerobic conditions, *C. jejuni* exhibited a substantial increase in the transcriptional levels of the antioxidant genes. The intracellular ATP levels were measured to examine the physiological activity of *C. jejuni* under aerobic conditions. The ATP concentrations were approximately 16-fold higher under microaerobic conditions than aerobic cultures after 24 h (Figure 1C), indicating that *C. jejuni* is not physiologically active under aerobic conditions. Microscopic observation with the LIVE/DEAD staining revealed that most viable *C. jejuni* cells were in coccoid forms under aerobic conditions (Figure 1D). Based on the morphology and ATP levels, *C. jejuni* is likely to enter a viable-but-non-culturable (VBNC) state under aerobic conditions.

### Abrogation of oxidant-mediated induction of *ahpC*, *katA*, and *sodB* by a *perR* mutation

**Figure 2:**
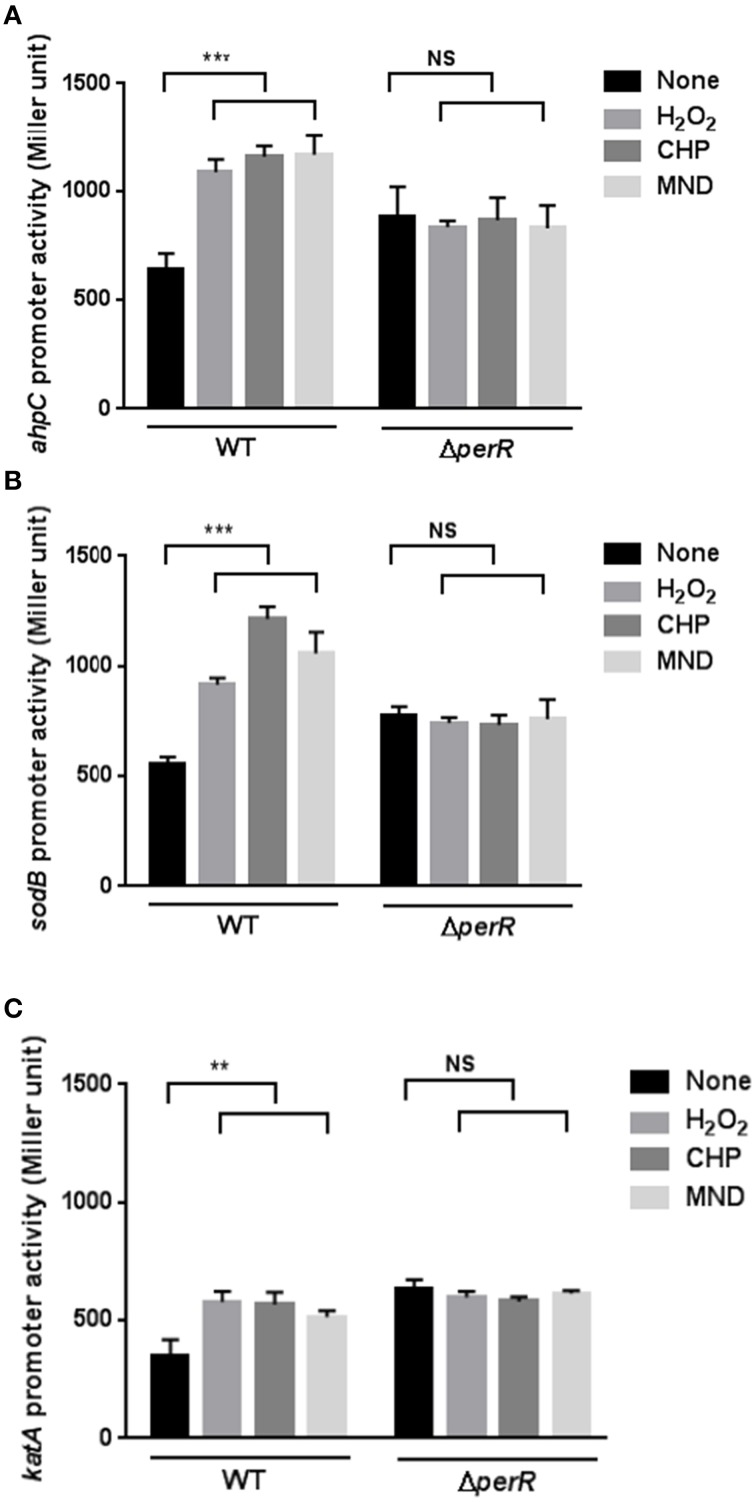
**Effects of a *perR* mutation on the transcription of *ahpC* (A), *sodB* (B), and *katA* (C) under oxidative stress**. β-Galactosidase assays of *ahpC*, *sodB*, and *katA* expression after exposure to 1 mM hydrogen peroxide (H_2_O_2_), 100 μM cumene hydroperoxide (CHP), and 100 μM menadione (MND). The promoter fusion assays were performed in both wild type and a *perR* mutant. After 8 h culture under microaerobic conditions, *C. jejuni* was exposed to oxidants for 2 h prior to an assay. The values represent the means and standard deviations of three independent experiments. Statistical analysis was performed with the Student's *t*-test using GraphPad Prism 6 (GraphPad Software Inc., USA). ^**^, *P* < 0.01; ^***^, *P* < 0.001; NS, not significant.

Since the transcriptional levels of *ahpC*, *katA*, and *sodB* were increased under aerobic conditions (Figure 1C) and the common biological role of the antioxidant genes is ROS detoxification, we hypothesized the augmented transcriptional levels in *ahpC*, *katA*, and *sodB* under aerobic conditions would result from increased oxidative stress in *C. jejuni*. Thus, we investigated whether oxidants would increase the promoter activity of the ROS-detoxification genes. The promoter activities of *ahpC*, *katA*, and *sodB* were monitored in the presence of oxidizing agents, such as H_2_O_2_, CHP (an organic peroxide), and MND (a superoxide anion generator). Regardless of the oxidant type, whether peroxide or superoxide, the transcription of *ahpC*, *katA*, and *sodB* was induced by oxidant exposure (Figure 2). The transcriptional induction by oxidants occurred in a concentration-dependent manner, and antioxidant treatment removed the oxidant-mediated induction of *ahpC*, *katA*, and *sodB* transcription (Supplementary Figure 1).

Since PerR is a major regulator of oxidative stress in *C. jejuni* (Van Vliet et al., 1999), we investigated if PerR could be associated with the transcriptional changes in the mutants under oxidative stress. Interestingly, the transcriptional induction of *ahpC*, *katA*, and *sodB* by oxidants completely disappeared in the *perR* mutant (Figure 2), indicating that PerR is associated with the transcriptional changes in *ahpC*, *katA*, and *sodB* under oxidative stress. In addition, the *perR* mutation increased the transcriptional levels of *ahpC*, *katA*, and *sodB* (Figure 2), suggesting that the *perR* mutation de-repressed the antioxidant genes, even including *sodB*. These results strongly suggest that PerR plays a role in the transcriptional control of both peroxide- and superoxide- detoxification genes in *C. jejuni* under oxidative stress conditions.

### PerR regulation of *sodB* transcription

**Figure 3:**
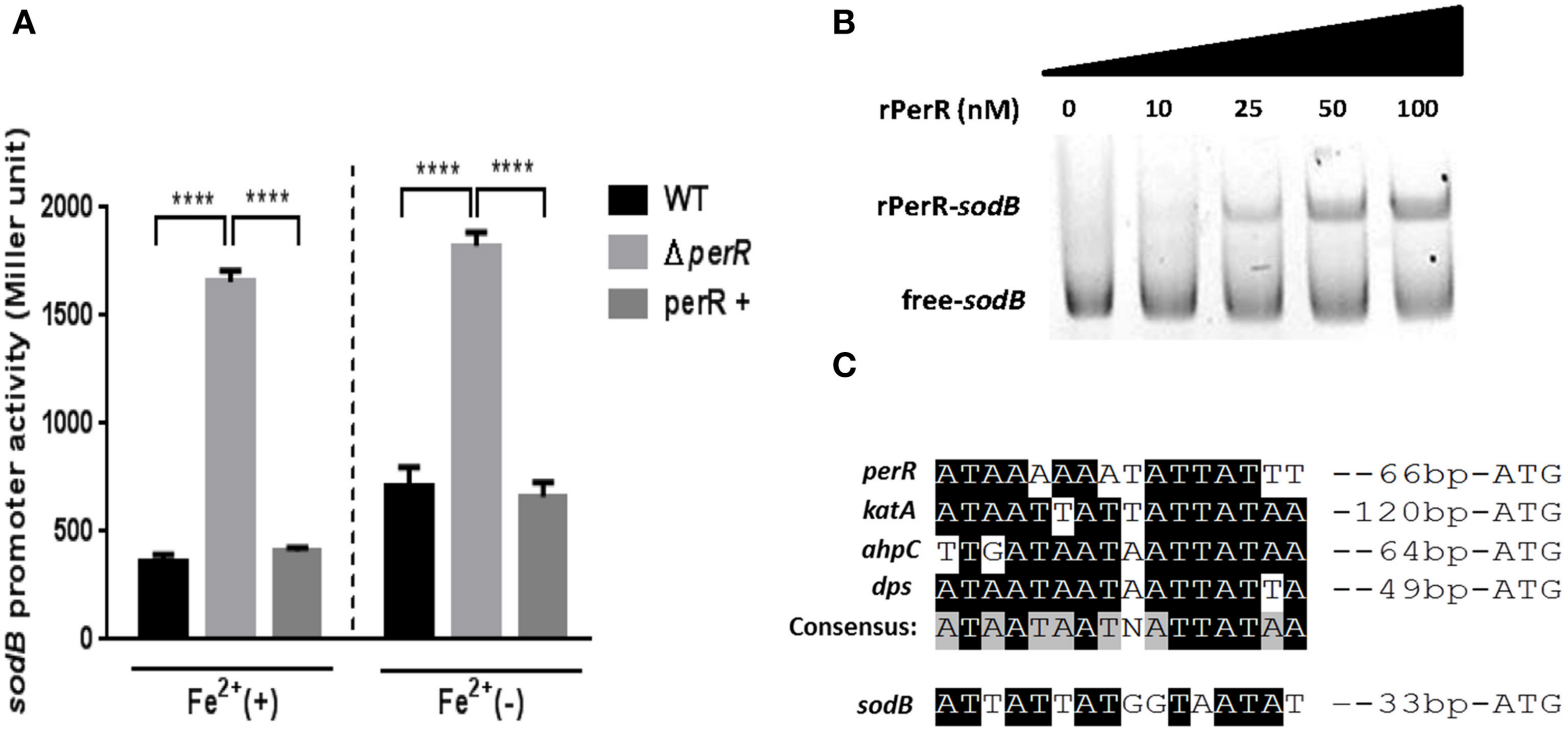
**PerR regulation of *sodB* in *C. jejuni***. **(A)** De-repression of *sodB* by a *perR* mutation. A *perR* mutation de-repressed *sodB* transcription, and the level of *sodB* transcription was completely restored by complementation. The *sodB* promoter activity was determined in MEMα with or without 40μM FeSO_4_. The results show the standard deviations of three different experiments. Statistical analysis was performed with a Student's *t*-test using GraphPad Prism 6. ^****^P < 0.0001; perR+, a *perR* complementation strain. **(B)** rPerR binding to the *sodB* promoter. Cy3-labeled PCR probes of a *sodB* promoter region were incubated with rPerR at different concentrations that are indicated above the gel. The assay was conducted in a 6% polyacrylamide gel under native conditions. **(C)** A putative PerR-binding site in the *sodB* promoter. The binding site was predicted based on the consensus PerR-binding sequence in *C. jejuni* (Kim et al., 2011).

Since a *perR* mutation affected *sodB* transcription (Figure 2B), we further investigated if PerR regulates *sodB* transcription by performing the *P_sodB_-lacZ* promoter fusion assay. A *perR* mutation de-repressed *sodB* transcription, regardless of the presence or absence of iron, and *perR* complementation restored *sodB* transcription to the wild-type level (Figure 3A). The increase in *sodB* transcription by the *perR* mutation was more significant in MEMα than MH broth (data not shown). To examine if PerR regulates *sodB* by binding to the *sodB* promoter, a gel-shift assay was performed with rPerR. Interestingly, rPerR directly bound to the *sodB* promoter (Figure 3B). A putative PerR binding site was predicted in the *sodB* promoter region based on the conserved PerR binding site in *C. jejuni* (Kim et al., 2011; Figure 3C). Taken together, these data suggest that PerR regulates *sodB* transcription by binding to the *sodB* promoter.

### Alteration of *perR* transcription by oxidants and aeration

**Figure 4:**
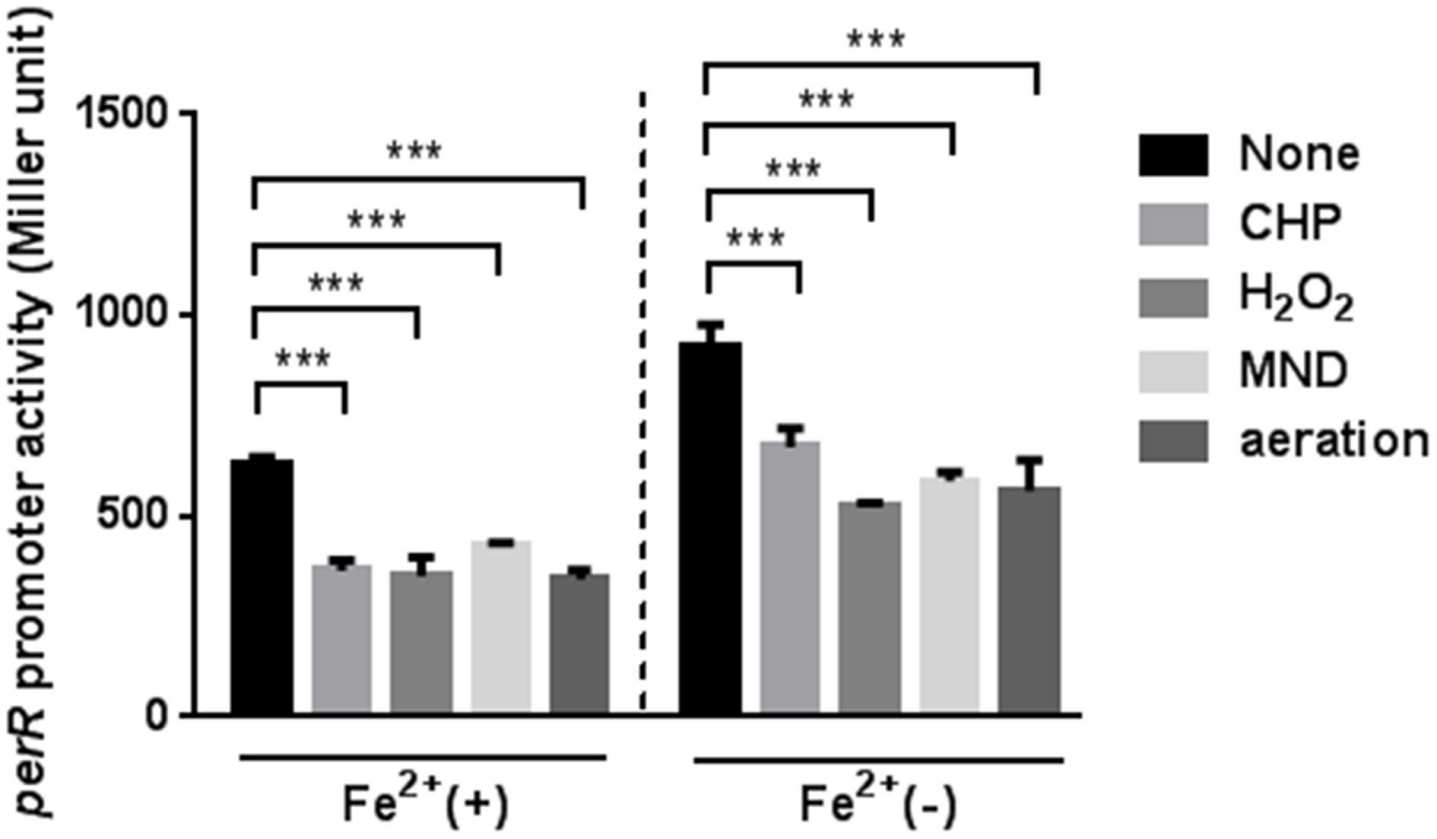
**Down-regulation of *perR* transcription under oxidative stress**. The *perR* promoter activity was evaluated after 2 h exposure to H_2_O_2_, CHP, MND, and aeration in MEMα with or without 40 μM FeSO_4_. The assays were repeated three times. Statistical analysis was performed with a Student's *t*-test. ^***^, *P* < 0.001.

Since the *perR* mutation completely removed the oxidant-mediated induction of *ahpC*, *sodB*, and *katA* (Figure 2), we investigated if *perR* transcription is affected by oxidants. The *P_perR_-lacZ* fusion assay was conducted with MEMα, because iron reduces *perR* transcription in *C. jejuni* (Kim et al., 2011). Oxidative stress, including oxidants (i.e., H_2_O_2_, organic peroxide, and superoxide) and aeration, substantially decreased the levels of *perR* transcription (Figure 4). The reduction of *perR* transcription by oxidative stress was not influenced by iron, although iron decreased the levels of *perR* transcription (Figure 4). The reduction of *perR* transcription by aeration would result from the increased ROS accumulation under aerobic conditions. The results clearly showed that *perR* transcription is affected by oxidative stress in *C. jejuni*.

## Discussion

PerR is a primary regulator of peroxide stress defense in many bacteria. In order for PerR to control the expression of peroxide stress defense genes, bacteria should control the intracellular level of PerR in response to peroxide stress. However, previous observations in other bacteria have shown that *perR* transcription is not affected by H_2_O_2_. For example, a transcriptomic analysis in *Staphylococcus aureus* demonstrated that the transcription of *perR* and its regulatory genes is not altered by H_2_O_2_ exposure (Chang et al., 2006). In *B. subtilis*, *perR* transcription is not changed by H_2_O_2_ (Fuangthong et al., 2002); instead, PerR regulates peroxide resistance genes by protein conformational changes under peroxide stress (Herbig and Helmann, 2001). *B. subtilis* PerR senses H_2_O_2_ by oxidation of one of two histidine residues (i.e., H37 and H91) that coordinate the bound Fe^2+^; this modification results in the dissociation of Fe^2+^ from PerR. The demetallated PerR cannot bind to DNA, and this conformational changes in PerR induces gene expression (Lee and Helmann, 2006). Interestingly, in this study, we demonstrated that oxidative stress, such as oxidant exposure and aerobiosis, directly reduced the levels of *perR* transcription (Figure 4). To the extent of our knowledge, *C. jejuni perR* is the only *perR* homolog whose transcription is known to be affected by oxidative stress. Although PerR is considered as a peroxide resistance regulator, *perR* transcription was altered by exposure to both peroxides (H_2_O_2_ and CHP) and superoxide (MND; Figure 4). Since PerR represses *ahpC* and *katA* (Van Vliet et al., 1999) and *sodB* (Figure 3), down-regulation of *perR* transcription under oxidative stress will de-repress *ahpC*, *katA*, and *sodB*. Consistently, we observed that the transcriptional levels of *ahpC*, *katA*, and *sodB* were increased by aeration and oxidant exposure (Figures 1, 2), and a *perR* mutation abrogated the transcriptional response of *ahpC*, *katA*, and *sodB* to oxidants (Figure 2), suggesting that PerR is a key player in the induction of both peroxide- and superoxide-detoxification genes under oxidative stress.

In this study, we demonstrated that PerR negatively regulates *sodB* expression (Figure 3). Based on the promoter sequence of *sodB* in *C. jejuni* (Pesci et al., 1994), a predicted PerR-binding site overlaps with the -10 region of the *sodB* promoter (data not shown); thus, PerR binding would interfere with *sodB* transcription. PerR is known as a regulator of peroxide resistance. However, our findings revealed that PerR also regulates *sodB* transcription in *C. jejuni*. To the best of our knowledge, this report is first describing direct regulation of *sodB* transcription by PerR. As a key enzyme of superoxide resistance, SOD catalyzes the dismutation of superoxide to H_2_O_2_. Due to its physiological importance in oxidative stress defense, bacteria often harbor redundant types of SOD. For example, *E. coli* contains three types of SOD, including two cytoplasmic SOD isoenzymes [SodA (Mn-cofactored SOD) and SodB (Fe-cofactored SOD)] and a periplasmic SOD [SodC (Cu/Zn-cofactored SOD)](Imlay, 2008). However, SodB is the sole SOD present in *Campylobacter* (Pesci et al., 1994; Purdy and Park, 1994), significantly contributing to *Campylobacter*'s stress resistance and colonization of chicken intestines (Pesci et al., 1994; Palyada et al., 2009). Various mechanisms for the regulation of *sod* genes have been reported in different bacteria. In *E. coli* and *S. enterica*, exposure to superoxide-generating agents increases SodA expression through positive regulation by SoxRS (Greenberg et al., 1990; Pomposiello et al., 2001); however, *C. jejuni* lacks the SoxRS system (Parkhill et al., 2000). In *E. coli*, Fur indirectly regulates *sodB* expression by RhyB small RNA (Masse and Gottesman, 2002), while apo-Fur represses *sodB* by directly binding to the *sodB* promoter in *Helicobacter pylori* (Ernst et al., 2005); however, *sodB* is not found in the Fur regulon of *C. jejuni* (Butcher et al., 2012). In *C. jejuni*, the *sodB* regulation relies on the two-component regulatory systems, such as CosR and CprS (Svensson et al., 2009; Hwang et al., 2011). In this study, we have demonstrated another novel mechanism of *sodB* regulation by PerR in *C. jejuni*.

Since *C. jejuni* is a microaerophilic bacterium, prolonged exposure to aerobic conditions substantially reduced the growth of *C. jejuni*; aerobic growth for 24 h resulted in an approximate 5-log reduction in colony forming units (Figure 1A). However, the transcriptional levels of *ahpC*, *katA*, and *sodB* were higher under aerobic conditions than microaerobic conditions (Figure 1B). *C. jejuni* exhibited aerotolerance but was not physiologically active based on the intracellular ATP levels (Figure 1C). In addition, the morphology of *C. jejuni* changed from spiral rods to coccoid forms under aerobic conditions (Figure 1D). A previous study demonstrated that *C. jejuni* is able to survive under prolonged exposure to aerobic stress by forming VBNC cells with a typical morphological change to coccoid forms (Rollins and Colwell, 1986). Harvey and Leach reported the formation of coccoid forms of *C. jejuni* is enhanced by high oxygen tension, presumably as a result of oxidative damage, and coccoid forms of *C. jejuni* may regain normal spiral morphology following adaptation to oxidative stress (Harvey and Leach, 1998). In response to various harsh stress conditions, many bacterial species are known to enter a VBNC state with significant dwarfing in size (Oliver, 2010). Although a few stress conditions have been reported to induce a VBNC state in *Campylobacter*, including nutrient starvation (e.g., incubation in water), cold stress (e.g., 4°C), and organic acids (e.g., formic acid) (Rollins and Colwell, 1986; Harvey and Leach, 1998; Kassem et al., 2013), mechanisms for the VBNC formation remain largely unknown. Based on the findings in this study, *C. jejuni* enters a VBNC state under aerobic conditions. Compared with microaerobic conditions, interestingly, the transcriptional levels of *ahpC*, *katA*, and *sodB* were higher under aerobic conditions, in which the majority of *C. jejuni* cells were in coccoid forms (Figures 1B,D). At this stage, it is not clear whether the up-regulation of *ahpC*, *katA*, and *sodB* occurred in viable-and-culturalble or VBNC cells or both. However, increased expression of the antioxidant genes will alleviate oxidative stress under aerobic conditions and may contribute to the protection of *C. jejuni* from increased oxidative stress during the physiological transition of *C. jejuni* to a VBNC state under aerobic conditions.

In this study, we presented a unique regulatory mechanism of oxidative stress defense in *C. jejuni* that non-selectively regulates superoxide and peroxide stress via PerR. Such an integrative regulatory system of PerR will help *C. jejuni* to survive in oxygen-rich conditions during transmission from food to humans by allowing for effective coordination of the expression of relatively few ROS-detoxification enzymes in this microaerophilic foodborne pathogen. Future studies will include the investigation of the role of the unique stress response mechanism in the survival of *C. jejuni* under stress (i.e., oxygen-rich) conditions, and the interplay between PerR and other oxidative stress response regulators in *C. jejuni*.

### Conflict of interest statement

The authors declare that the research was conducted in the absence of any commercial or financial relationships that could be construed as a potential conflict of interest.
