# Supplementary material for: Non-selective regulation of peroxide and superoxide resistance genes by PerR in Campylobacter jejuni
Source: Front Microbiol. 2015 Feb 17;6:126. doi: 10.3389/fmicb.2015.00126 (PMC4330884; doi:10.3389/fmicb.2015.00126)
Supplement: Supplementary file 1 [file Presentation1.PDF]

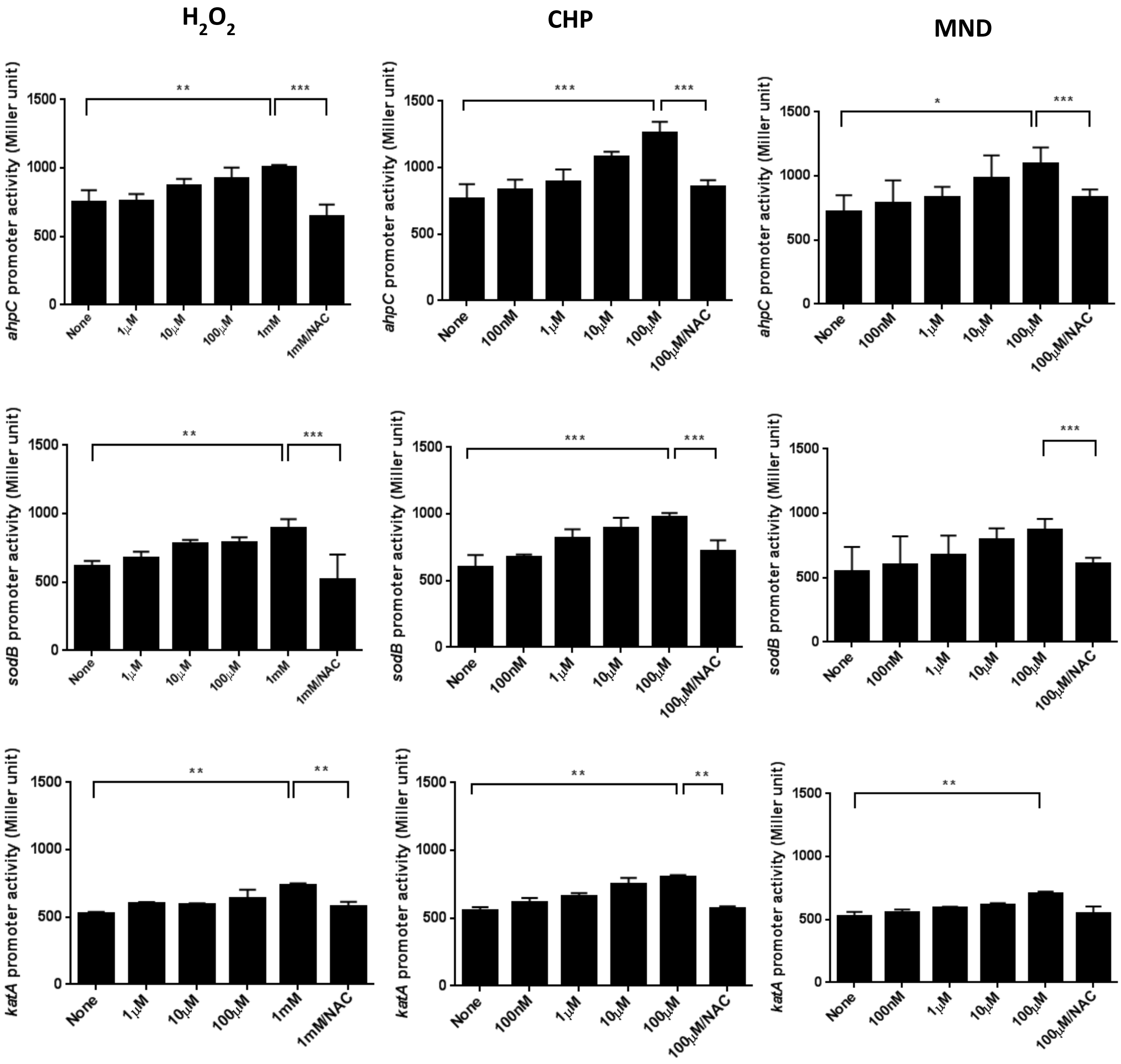

Supplementary Fig. 1. Does-dependent induction of *ahpC, sodB*, and *katA* transcription by oxidants. β-Galactosidase assays of *ahpC*, *sodB*, and *katA* expression after exposure to hydrogen peroxide (H_2O_2), cumene hydroperoxide (CHP), and menadione (MND) at the indicated concentrations. NAC was added to a final concentration of 1 nM. Exponentially grown *C. jejuni* cells were collected and treated with oxidants for 2 h. The results show the means and standard deviations of three different experiments. The statistical analysis was conducted with Student's *t*-test *: *P*<0.1; **, *P*<0.01; ***, *P*<0.001.
